# 

**Published:** 1895-10-19

**Authors:** 


### CONTENTS.

**The Three Congresses: Their Lessons for Medicine** - 39

**Dr. J. S. Billings** - 40

**On Modern Progress in Ophthalmic Medicine and Surgery** By R. B. Carter, F.R.C.S.—Squint (*continued*) - 41

**Social Problems.**—Medicine and Pauperism in Country Districts 42

**Medical Progress and Hospital Clinics—**

The Physiology of Œdema. By G. Munro Smith - 45

The Schott Treatment for Heart Disease. By Richard Greene, F.R.C.P.E. - 46

Progress in Laryngology (*continued*) - 49

**Annotations.**—Large or Small Families?—The Current that Kills—The Health of Shanghai - 43

**Notes and News** - 44

**The Institutional Workshop—**

Hospital Expenditure.—The Commissariat. III.—The Question of Numbers - 51

Practical Departments.—Furniture and Fittings for Nurses' Homes.—II. - 52

Hospital and Asylum Construction - 52

**Scraps and Gleanings** - 53

### EXTRA SUPPLEMENT.—THE NURSING MIRROR.

**News from the Nursing World.**—A Brave Nurse Rewarded—Nurses at the Temperance Hospital—Our Needlework Competition—Nurses at Greenwich Union—The Whittuck Convalescent Home—A Cottage Hospital for Accrington—A Breach of Dicipline at Lincoln—Nursing at Barry Dock—Gainsborough Nursing Association—Victoria Home, Cheltenham—Night Nursing at Coventry—Forfar District Nursing Association—Queen's Nurses at Aberdeen—An American Door Porter—Short Items - xix
**Elementary Physiology for Nurses.** By C. F. Marshall, late Surgical Registrar, Hospital for Sick Children, Great Ormond Street. XI.—The Digestive System (*concluded*) xxi
**Notes and Queries** - xxi
**Novelties for Nurses** - xxii
**Neuralgia and Its Treatment.—II.** - xxii
**Where to Go—Minor Appointments** - xxiii
**Our American Letter** - xxiii
**Everybody's Opinion** - xxiv

